# Personalized circulating tumor DNA detection to monitor immunotherapy efficacy and predict outcome in locally advanced or metastatic non‐small cell lung cancer

**DOI:** 10.1002/cam4.6108

**Published:** 2023-05-15

**Authors:** Lei Cheng, Guanghui Gao, Chao Zhao, Haowei Wang, Chao Yao, Hanchuanzhi Yu, Jichen Yao, Feng Li, Lijie Guo, Qijie Jian, Xiaoxia Chen, Xuefei Li, Caicun Zhou

**Affiliations:** ^1^ Department of Lung Cancer and Immunology, Shanghai Pulmonary Hospital, School of Medicine Tongji University Shanghai China; ^2^ Department of Medical Oncology, Shanghai Pulmonary Hospital, School of Medicine Tongji University Shanghai China; ^3^ OrigiMed Co., Ltd Shanghai China

**Keywords:** circulating tumor DNA, immune checkpoint inhibitors, monitoring efficacy, non‐small cell lung cancer, outcome prediction

## Abstract

**Objective:**

Immune checkpoint inhibitors (ICIs) or combined with chemotherapy exhibit substantial efficacy for the treatment of advanced non‐small cell lung cancer (NSCLC). However, reliable biomarkers that can monitor response to first‐line ICIs ± chemotherapy remain unclear.

**Methods:**

A total of 16 tumor tissues and 46 matched peripheral blood samples at baseline and during treatment were retrospectively collected from 19 locally advanced or metastatic NSCLC patients. The circulating tumor DNA (ctDNA) burden by tumor‐informed assay was detected to monitor and predict the therapeutic response and survival of NSCLC patients treated with first‐line ICIs or plus chemotherapy.

**Results:**

We found that ctDNA was only positively detected in one patient by tumor‐agnostic assay with a mean variant allele fraction (VAF) of 6.40%, whereas it was positively detected in three patients by tumor‐informed assay with a mean VAF of 8.83%, 0.154%, and 0.176%, respectively. Tumor‐informed assays could sensitively detect ctDNA in 93.75% (15/16) of patients. Trends in the level of ctDNA from baseline to first evaluation was consistent with the radiographic changes. There was a greater decrease in ctDNA after treatment compared with baseline in patients with partial response compared to patients with stable disease/progressive disease. Patients with over a 50% reduction in ctDNA had a significant progression‐free survival and overall survival benefit.

**Conclusion:**

The tumor‐informed assay was favorable for ctDNA detection, and early dynamic changes in plasma ctDNA may be a valuable biomarker for monitoring the efficacy and predicting the outcome in advanced NSCLC patients treated with first‐line ICIs ± chemotherapy.

## 1 INTRODUCTION

Immune checkpoint inhibitors (ICIs) targeting programmed cell death protein 1 (PD‐1) or programmed cell death ligand 1 (PD‐L1) in combination with chemotherapy represent the standard of care for patients with driver‐gene negative and advanced non‐small cell lung cancer (NSCLC). Although some patients can produce remarkably durable responses, most develop early disease progression.^1^, ^2^, ^3^ PD‐L1 is limited as a biomarker for predicting a response to treatment with ICIs plus chemotherapy. Other biomarkers, such as the tumor mutational burden (TMB), have not been incorporated into routine clinical practice for treatment selection.^4^, ^5^ In addition, an initial assessment by CT imaging is often unable to identify which patients will achieve a durable clinical benefit. Furthermore, reliable biomarkers capable of accurately identifying the efficacy of immunotherapy remain elusive.

Circulating tumor DNA (ctDNA) is a component of cell‐free DNA (cfDNA), released from apoptotic or necrotic tumor cells.^6^ Plasma ctDNA has been shown to reflect the mutational signatures of the primary tumor and is emerging as a potential noninvasive biomarker for evaluating tumor burden and response to treatment.^7^, ^8^ Several studies have indicated a clinical utility of ctDNA for molecular residual disease (MRD) assessment, monitoring recurrence, and the treatment response in NSCLC patients.^9^, ^10^ Previous studies also have shown that dynamic ctDNA changes constitute a valuable predictive tool for the treatment response, progression‐free survival (PFS) and overall survival (OS) in patients treated with immunotherapy.^11^, ^12^, ^13^, ^14^, ^15^, ^16^, ^17^, ^18^, ^19^ ctDNA dynamics can be measured by next‐generation sequencing (NGS) technology, including tumor‐informed assay and tumor‐agnostic assay. The former is a patient‐specific panel designed based on whole‐exome sequencing (WES), whereas the latter is a fixed panel. All of the above studies were based on tumor‐agnostic assay and included NSCLC patients treated by ICIs as a second line or later treatment. A recent study suggested that a tumor‐informed panel outperforms a tumor‐agnostic panel for monitoring MRD in resected NSCLC.^20^ However, whether ctDNA can predict the effect of first‐line immunotherapy in patients with locally advanced or metastatic NSCLC, especially by tumor‐informed assay, remains unclear. Therefore, we conducted a retrospective study to evaluate whether ctDNA could be used as a biomarker to identify NSCLC patients who may benefit from immunotherapy and to track the treatment response.

## 2 MATERIALS AND METHODS

### 2.1 Patients and sample collection

A total of 19 locally advanced or metastatic NSCLC patients, who received first‐line ICI monotherapy or combined with chemotherapy were retrospectively enrolled in our study. Tumor tissue samples and matched peripheral blood samples were collected from 16 patients at baseline. At least two serial blood samples at baseline and at the beginning of second/third cycle were obtained for all 19 patients. Clinical data, including age, gender, smoking status, tumor subtype and stage were collected. Clinical responses, including partial response (PR), stable disease (SD) and progression disease (PD) were evaluated based on RECIST 1.1 criteria. PFS was characterized as the time interval from the initiation of treatment to the date of disease progression or death. OS was defined as the time interval from the initiation of treatment to the date of death or last follow‐up. This study was approved by the Ethical Committee of Shanghai Pulmonary Hospital with reference number K20‐288, and all patients provided informed consent.

### 2.2 DNA extraction and purification

Genomic DNA (gDNA) was extracted from the formalin‐fixed, paraffin‐embedded (FFPE) tumor samples and matched white blood cells (WBCs) using a QIAamp DNA FFPE Tissue Kit (QIAGEN) and a QIAamp DNA Blood Mini Kit (QIAGEN), respectively in accordance with the manufacturer's instructions. Plasma cfDNA was purified using a QIAamp Circulating Nucleic Acid Kit (QIAGEN) according to the manufacturer's protocol.

### 2.3 Whole‐exome sequencing

Qualified gDNAs were randomly fragmented, and the quality and size of the fragments were determined using a LabChip GX Tough Nucleic Acid Analyzer (PerkinElmer), and were subsequently used for library preparation and sequencing in accordance with the manufacturer's instructions. Whole‐exome sequencing (WES) was constructed using xGen™ Exome Hyb Panel V2 (Origimed), and the captured DNA library was sequenced on an Illumina NovaSeq 6000 sequencer (Illumina) with a mean depth of 500×. Library construction and sequencing were performed in a Clinical Laboratory Improvement Amendments (CLIA)/College of American Pathologists (CAP) compliant Molecular Diagnostics Service Laboratory of Shanghai OrigiMed Co., Ltd. The clean reads acquired using FASTP^21^ were mapped onto human genome hg19 reference sequences, and duplicate reads were removed with Picard after converting the format. Single nucleotide variants (SNVs), insertion‐deletions, copy number variation regions, gene fusions, and gene rearrangements were analyzed using MuTect (v1.7),^22^ PINDEL (v0.2.5),^23^ Control‐FREEC (v9.7),^24^ an in‐house developed algorithm and integrative genomics viewer, respectively. The functional impact of the genomic alterations was annotated by SnpEff3.0.^25^ The results were annotated to several databases, including the Reference Sequence (Refseq), 1000 Genomes, Genome Aggregation Database (gnomAD), the Exome Aggregation Consortium (ExAC), NHLBI GO Exome Sequencing Project 6500 (ESP6500), Sorting Intolerant from Tolerant (SIFT), PolyPhen, and Catalog of Somatic Mutations in Cancer (COSMIC) databases. By comparing the tumor tissues with matched WBC samples, germline mutations were filtered out and only somatic mutations were retained during the analysis.

### 2.4 Tumor‐informed assay

Design and application of personalized ctDNA detection using a tumor‐informed assay (OriMIRACLE S™) was conducted with blinding to clinical data by OrigiMed. OriMIRACLE S™ can stably detect mutations with variant allele fraction (VAF) ≥0.02% for monitoring therapeutic efficacy.^26^ Tumor tissues and matched peripheral blood were collected before treatment and WES was prepared. For each patient, we selected up to 30 clonal somatic mutations for a personalized ctDNA assay design. Serial plasma samples at baseline and at the beginning of treatment were performed with OriMIRACLE S™.

### 2.5 Tumor‐agnostic assay

Tumor‐agnostic assay was performed using a 671‐gene panel associated with cancer diagnostics, followed by NGS with an average sequencing depth of approximately 15,000×, noise filtering and molecular tracking, and variant calling for SNVs. Tumor‐informed assay and tumor‐agnostic assay were performed simultaneously in three stage IV patients. The ctDNA burden (hGE/mL) was calculated as mean mutant tumor molecules per milliliter of plasma based on mean VAF and cfDNA yield according the following equation^27^:

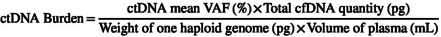




### 2.6 Statistical analysis

Statistical analysis was performed using R Statistical Software package (R Foundation for Statistical Computing). For all statistical analyses, ctDNA VAF for each sample was defined as the mean VAF of the detected ctDNA alterations. A Student's *t*‐test or Wilcoxon rank test was used to compare two continuous data sets. Spearman's correlation coefficients were used to analyze the relationship between ctDNA VAF and tumor measurements. A swimmer plot was performed to visualize the clinical response and ctDNA changes for each patient. Kaplan–Meier curves were carried out to assess the effect of ctDNA on OS and PFS. A two‐sided *p* < 0.05 was considered to be statistically significant.

## 3 RESULTS

### 3.1 Patient characteristics

**FIGURE 1:**
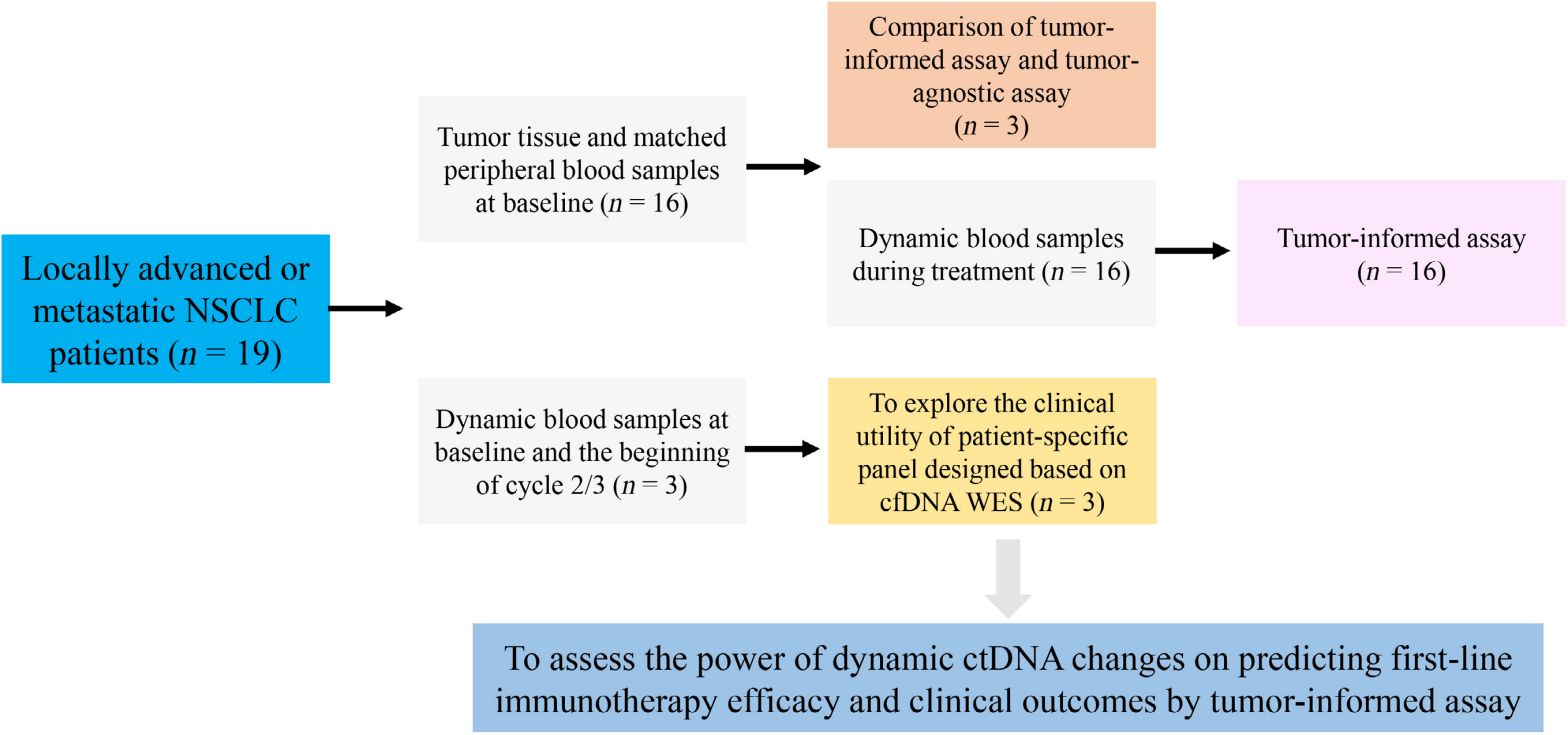
Workflow of the study. cfDNA, cell‐free DNA; ctDNA, circulating tumor DNA; NSCLC, non‐small cell lung cancer; WES, whole‐exome sequencing.

**FIGURE 2:**
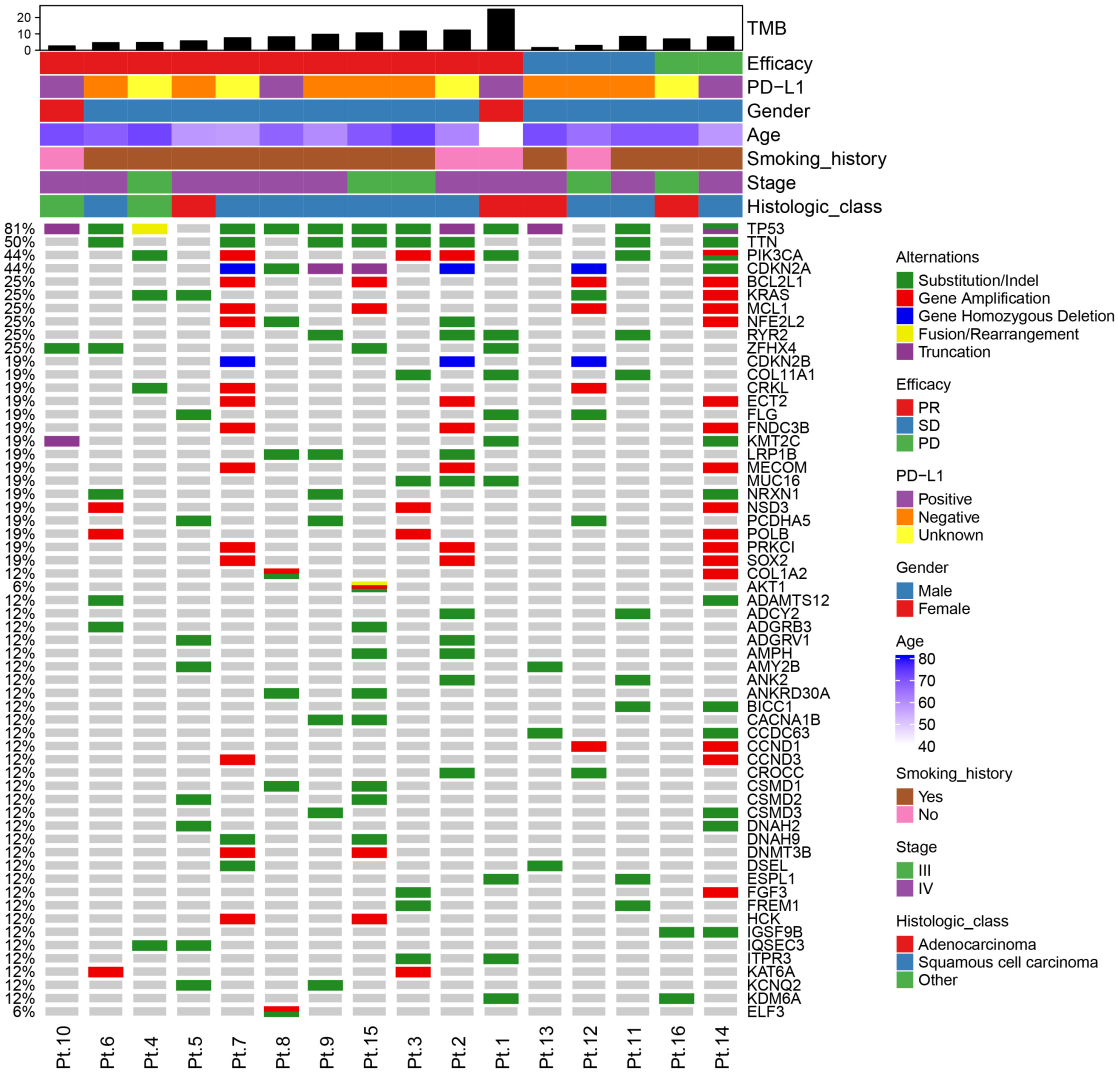
Mutations detected by WES in tissues at baseline from 16 patients with locally advanced or metastatic NSCLC. The rows at the top show TMB level, clinical responses, PD‐L1 expression level (including 22C3 and E1L3N), gender, age, smoking history, stage and pathologic type. The rows at the middle indicate mutated genes ordered based on decreasing prevalence. NSCLC, non‐small cell lung cancer; PD, progression disease; PD‐L1, programmed cell death ligand 1; PR, partial response; SD, stable disease; TMB, tumor mutational burden; WES, whole‐exome sequencing.

A total of 19 locally advanced or metastatic NSCLC patients were enrolled in this study (Figure 1). The patient characteristics are shown in Table S1. The cohort included more men (*n* = 17, 89.5%) than women (*n* = 2, 10.5%) ranging in age from 40 to 73 years. Among these patients, 14 (73.7%) were Stage IV and 5 (26.3%) were Stage III. There were 15 (78.9%) patients with a smoking history and 4 (21.1%) patients with no smoking history. Of the 19 patients, 1 (5.3%) patient had brain metastasis, 2 (10.5%) patients had liver metastasis, and 5 (26.3%) patients had bone metastasis. The patients received ICI treatment, including pembrolizumab monotherapy (15.8%), pembrolizumab plus chemotherapy (68.4%) and other ICIs plus chemotherapy (15.8%). There were 12 (63.2%) patients who achieved PR, 5 (26.3%) SD, and 2 (10.5%) PD. Figure 2 shows the mutational profiling of the 16 patients at baseline. The most frequently altered genes included TP53 (81%), TTN (50%), PIK3CA (44%), CDKN2A (44%), BCL2L1 (25%), KRAS (25%), MCL1 (25%), NFE2L2 (25%), RYR2 (25%) and ZFHX4 (25%).

### 3.2 Comparison of the ctDNA detection analysis between the tumor‐informed assay and tumor‐agnostic assay

Tumor‐informed assay and tumor‐agnostic assay were simultaneously performed in three patients with Stage IV NSCLC (Table 1). The detection results showed that the ctDNA was positively detected in only one patient by tumor‐agnostic assay, whereas it was positively detected in three patients using tumor‐informed assay. A total of 218, 301, and 225 SNVs were detected in the tumor tissues from three patients using WES. A total of 20 and 26 SNVs were detected in the plasma at baseline using tumor‐informed assay; however, no SNVs were observed using tumor‐agnostic assay for patients 8 and 2, respectively. To more accurately quantify low levels of ctDNA, the mean VAF (mVAF) from all tested variants were calculated, and we observed mVAF as 0.154% and 0.176% for patients 8 and 2, respectively, using tumor‐informed assay, whereas ctDNA was not detected by tumor‐agnostic assay. There were 29 SNVs detected using tumor‐informed assay with a mVAF of 8.83%, and 2 SNVs were observed by tumor‐agnostic assay with a mVAF of 6.40% for patient 11. These results suggest that the lower limit of VAF detection was the percentage level by tumor‐agnostic assay; however, it can be as low as a thousandth percentile using tumor‐informed assay.

**TABLE 1:** Comparison of the ctDNA detection analysis between tumor‐informed assay and tumor‐agnostic assay.

Patients	SNVs detected by WES	Tumor‐informed assay		Tumor‐agnostic assay	
		SNVs	VAF	SNVs	VAF
Pt.8	218	20	0.154%	0	0
Pt.2	301	26	0.176%	0	0
Pt.11	225	29	8.83%	2	6.40%

Abbreviations: ctDNA, circulating tumor DNA; SNV, single nucleotide variant; VAF, variant allele fraction; WES, whole‐exome sequencing.

### 3.3 ctDNA levels, clinical response, and radiographic response

**FIGURE 3:**
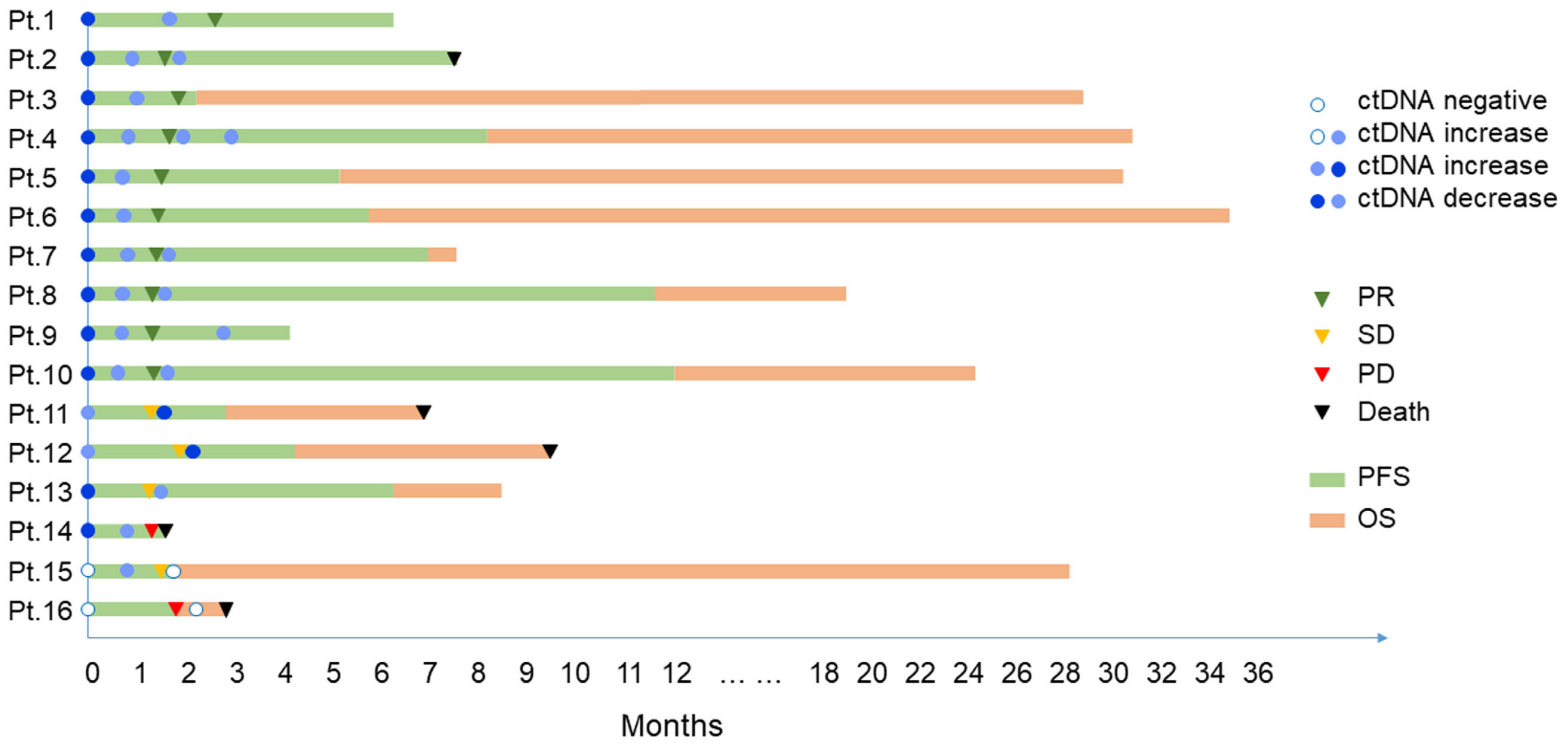
ctDNA changes during treatment. Event chart showing timing of therapy, clinical responses, outcomes, and results of ctDNA testing for each patient. ctDNA, circulating tumor DNA. PR, partial response. SD, stable disease. PD, progression disease. PFS, progression‐free survival. OS, overall survival.

We analyzed a total of 40 plasma samples from 16 patients at baseline and during treatment. Of these 16 patients, ctDNA was negatively detected in two patients and positively observed in the other patients at baseline. There were 10 out of 16 (62.5%) patients with decreased ctDNA that achieved PR (Pt.1–Pt.10); however, 2 out of 16 patients with decreased ctDNA achieved SD and PD, respectively (Pt.13 and Pt.14). Three patients (Pt.11, Pt.12, and Pt.15) had an increased ctDNA burden, all of whom achieved SD. One patient (Pt.16) who was tested negative for ctDNA both at baseline and after treatment developed PD and died (Figure 3).

In addition, changes in the ctDNA during treatment predicted an earlier clinical response. For patients with PR, ctDNA changes often preceded the radiographic response (Figure S1A–J). For patients who achieved SD, ctDNA changes were consistent with the radiographic response (Figure S1K–M).

### 3.4 Association between early changes in ctDNA levels with the clinical response

**FIGURE 4:**
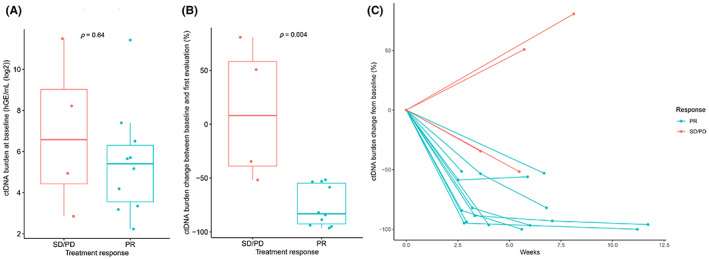
ctDNA changes predict benefit from first‐line immunotherapy. (A) ctDNA burden at baseline according to clinical responses. (B) ctDNA burden changes from baseline to first evaluation according to clinical responses. (C) ctDNA burden changes over time in patients with PR or SD/PD. ctDNA, circulating tumor DNA; PD, progression disease; PR, partial response; SD, stable disease.

We next explored whether early dynamic changes in the ctDNA levels had predictive value for the benefit of first‐line ICIs ± chemotherapy. Baseline ctDNA levels were not significantly different between patients with SD/PD and patients with PR (*p* = 0.64, Figure 4A). Changes in the ctDNA burden between baseline and the first evaluation after treatment were significantly greater in patients with PR than in those with SD/PD (*p* = 0.004, Figure 4B). Patients with PR exhibited a 75.68% decrease in the ctDNA burden from baseline, whereas patients with SD/PD showed an 11.37% increase (Figure 4C). Among the 14 patients with positive baseline ctDNA, the PR patients showed a greater decrease in ctDNA from baseline compared to patients with SD/PD. In addition, we explored a patient‐specific panel designed based on cfDNA WES, and observed a decrease in the ctDNA levels from baseline to first evaluation in patients with SD/PR (Figure S2 and Table S2).

### 3.5 Survival benefit in patients with over a 50% reduction in ctDNA levels

**FIGURE 5:**
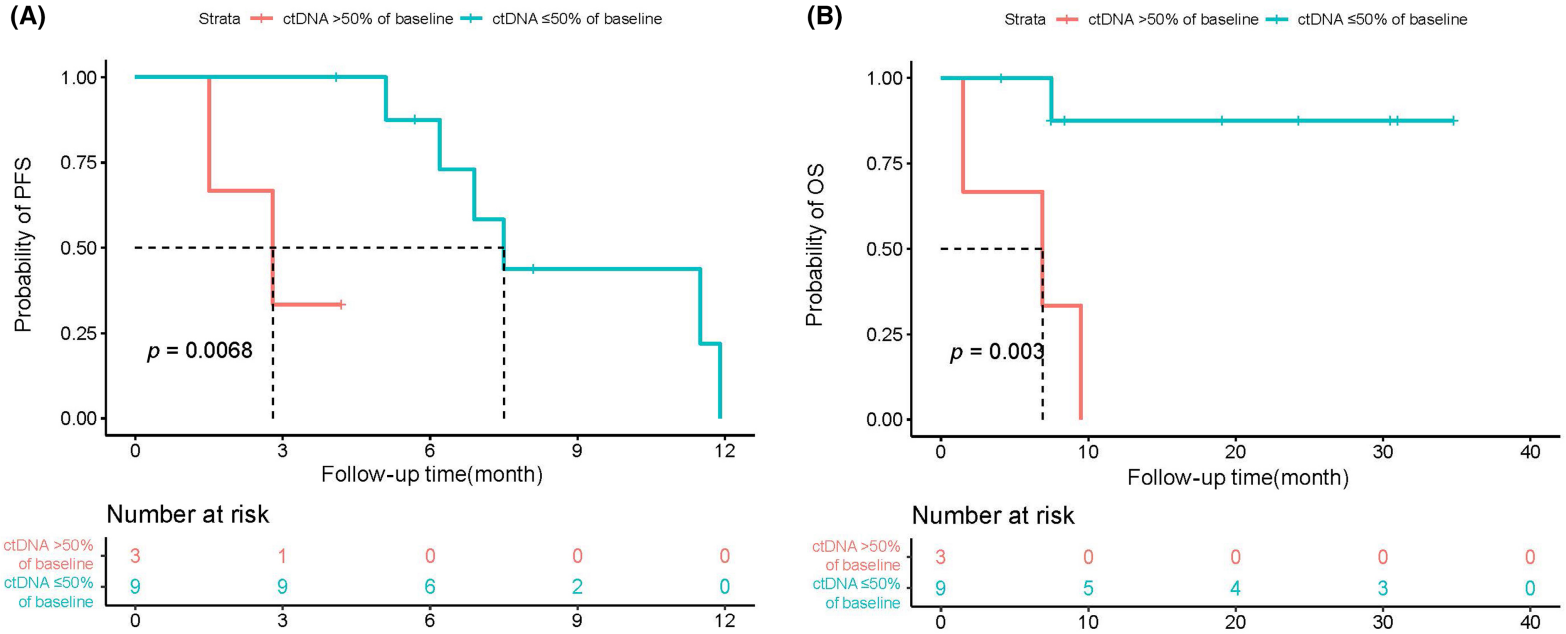
The changes in ctDNA levels over a 50% reduction from baseline are strongly correlated with PFS and OS. Kaplan–Meier curves for PFS (A) or OS (B) dichotomized using a 50% decrease in ctDNA burden relative to baseline. ctDNA, circulating tumor DNA; OS, overall survival; PFS, progression‐free survival.

Among the 12 patients with survival data, patients with over a 50% reduction at the first evaluation after treatment compared with baseline had a significant PFS and OS benefit (Figure 5A,B). In addition, the relationship between PD‐L1 expression with prognosis was analyzed and no significant correlation was found (Figure S3A,B). There was also no correlation observed between the TMB level and PFS. Patients with TMB below median exhibited a better OS than patients with TMB above median; however, there was no significant association (Figure S3C,D).

### 3.6 ctDNA predicts the response to first‐line pembrolizumab monotherapy in locally advanced NSCLC

**FIGURE 6:**
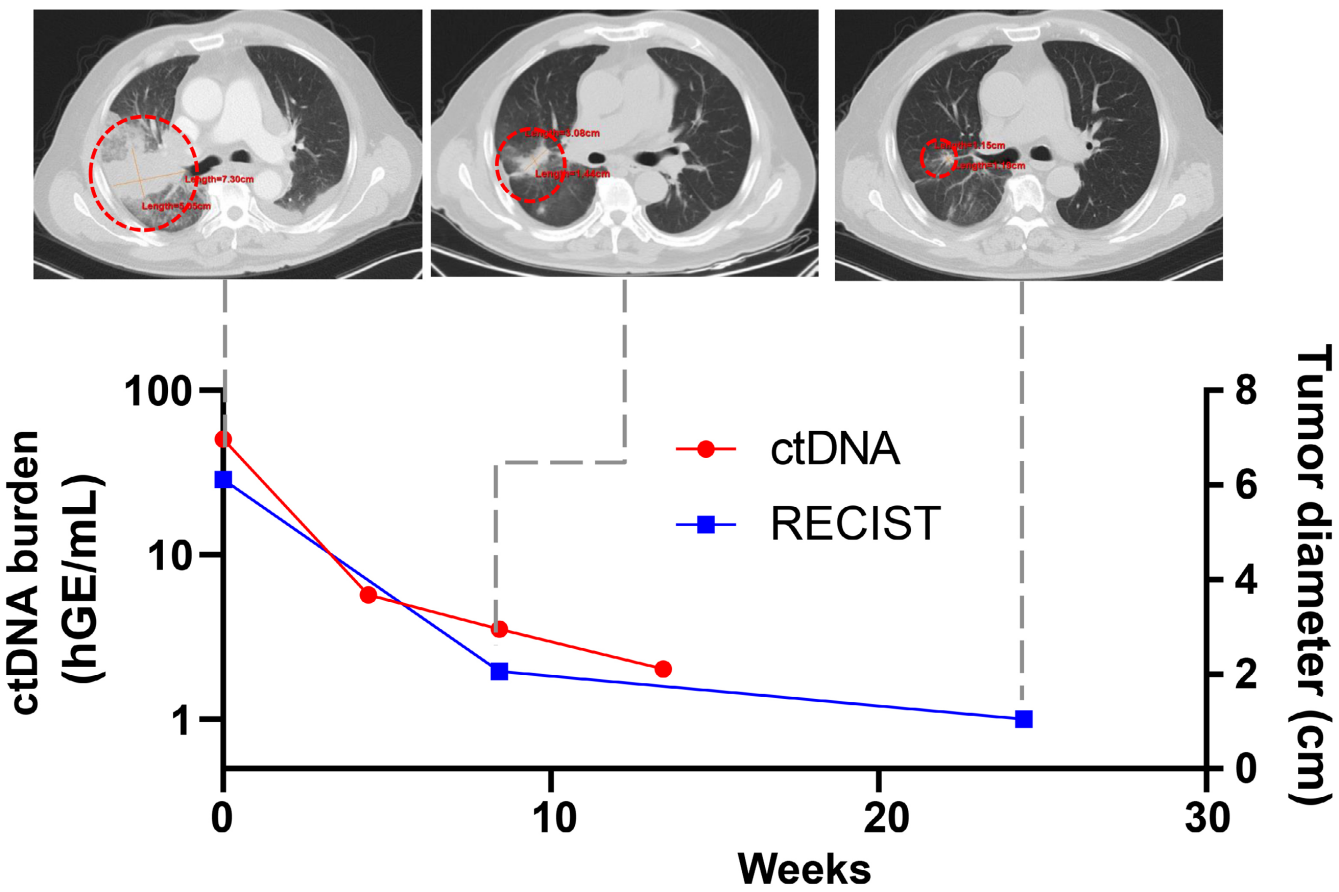
A patient case showed ctDNA dynamics and CT images at different time points during treatment. The 72‐year‐old man received six cycles of first‐line pembrolizumab monotherapy. The ctDNA burden showed an 88.63% decrease from baseline to Day 30 while the patient achieved PR on Day 59.

A 72‐year‐old patient was diagnosed with Stage IIIB NSCLC and received six cycles of first‐line pembrolizumab monotherapy. The ctDNA burden showed an 88.63% decrease from 50.20 hGE/mL at baseline to 5.71 hGE/mL on Day 30. The patient achieved PR on day 59 and the ctDNA changes functioned as an indicator of immunotherapy efficacy 1 month earlier than radiographic assessment (Figure 6).

## 4 DISCUSSION

Tumors release ctDNA into peripheral blood, which can be detected and quantified in liquid biopsies to monitor disease progression and response to treatment.^28^ Although studies have proposed a rationale for early ctDNA changes as an additional and potentially early assessment of the response of NSCLC to immunotherapy, these data were generated in a heterogeneous patient population that received ICIs primarily as second‐line or subsequent therapy.^12^, ^13^, ^14^, ^15^, ^16^, ^17^, ^18^ Therefore, whether early ctDNA dynamics could also predict the response to first‐line ICIs ± chemotherapy in patients with treatment‐naïve NSCLC remains unknown. In our study, patients with locally advanced or metastatic NSCLC receiving first‐line immunotherapy alone or combined with chemotherapy were enrolled in our study. We hypothesized that rapid changes in plasma ctDNA measured after treatment could enable the early detection of response to immunotherapy in advanced NSCLC.

NGS approaches have become prevalent for tumor sequencing and have also been applied to cfDNA for ctDNA detection.^29^ With the development of multiple platforms, including tumor‐informed and tumor‐agnostic ctDNA assays, it demonstrates their provocative analytic performance to detect ctDNA. A tumor‐informed assay was designed for ctDNA detection based on the genomic profiling of tumor tissue to identify tumor‐derived alterations specific for each individual patient. A personalized, tumor‐informed assay (Signatera, Natera) for ctDNA detection has been marketed for use in the surveillance of resected colorectal cancer.^30^, ^31^, ^32^ A tumor‐agnostic ctDNA assay is a blood‐based ctDNA assay that is not dependent a priori on tumor tissue profiling, including Guardant Reveal (Guardant Health).^33^, ^34^, ^35^ A recent study has indicated that a tumor‐informed assay is optimal over a tumor‐agnostic assay for risk evaluation in postoperative NSCLC patients.^20^ In our study, both tumor‐informed assay and tumor‐agnostic assay were performed to analyze the susceptibility for detecting ctDNA in three patients. We observed that the ctDNA was positively detected in only one patient by tumor‐agnostic assay with a mVAF of 6.40%, whereas there was no detection in the other two patients who were positively detected by tumor‐informed assay with mVAF of 0.154% and 0.176%, respectively. Moreover, ctDNA was positively detected at baseline in 14 of 16 patients (87.5%) by tumor‐informed assay in our study, whereas ctDNA was only positively detected at baseline in 57.1%–77.9% patients by tumor‐agnostic assay.^11^, ^12^, ^13^, ^14^, ^15^ In addition, we found that only 1–7 of mutations detected by WES were covered by a commercial 671‐gene panel and most mutations were not covered (Figure S4A). Moreover, 30 clonal somatic mutations selected for tumor‐informed assay design were almost not covered by a commercial 671‐gene panel and only 1–3 mutations in 8 out of the 16 patients were covered (Figure S4B). This can be explained by the fact that the commercial ctDNA big panels are usually designed to only detect the hotspot mutations in tumors, which guide treatment. WES detected mutations may not be hotspot mutations or driver mutations and are not covered by the fixed panel. Moreover, the fixed panel‐covered hotspot mutations may only be sub‐clonal mutations, which have lower chance to be present in the circulation, and this can be overcame by using WES dependent personalized panel design targeting clonal mutations. Taken together, the tumor‐informed assay as personalized ctDNA detection was found to be a better option compared to the tumor‐agnostic assay, had a high detection rate, and could maximize patient benefits.

ctDNA dynamics have been explored as a potential biomarker for the response to ICIs. Previous studies have shown that melanoma patients with no detectable levels of ctDNA prior or during treatment with ICIs have a better response to immunotherapy.^12^, ^36^ In NSCLC, patients with undetectable ctDNA posttreatment with ICIs displayed improved survival compared with patients with detectable ctDNA.^14^ A response evaluation based on ctDNA remains a complex issue without standardization, which thus, requires further clinical exploration. Herein, we found that ctDNA changes were consistent with the radiographic response. Moreover, decreases in ctDNA often preceded the radiographic response for patients with PR. Although Pt.14 who underwent a left pneumonectomy 2 years ago showed a ctDNA decrease but achieved PD, the ctDNA burden only showed a 34.55% reduction from 2854.06 hGE/mL to 1868.07 hGE/mL. The patients with PR exhibited a 75.68% decrease in the mean ctDNA burden from baseline levels, whereas patients with SD/PD showed an 11.37% increase in the mean ctDNA burden from baseline. A decrease in ctDNA following treatment compared with baseline was greater in patients with PR compared to patients with SD/PD. In our study, patients with over a 50% reduction in the first evaluation after treatment had a significant PFS and OS benefit. It has also been previously reported that patients whose ctDNA levels decreased by ≥50% exhibited a longer benefit compared with those whose ctDNA decreased by <50% in NSCLC.^14^, ^15^ Together, these findings suggest that the variation tendency in ctDNA before and after immunotherapy may be a valuable biomarker for outcome prediction in advanced NSCLC patients.

Since the tumor‐informed assay was dependent on tissue WES, we explored a patient‐specific panel designed based on cfDNA WES. A total of 27, 14, and 37 SNVs from three patients were selected as a personalized panel, respectively. A total of 26, 14, and 37 SNVs were successfully detected during treatment and there was an observed decrease in the levels of ctDNA from baseline to first evaluation in patients with SD/PR. It is suggested that blood‐informed liquid biopsy may represent a potentially feasible approach for monitoring efficacy in tissue‐unavailable patients; however, further exploration and verification of multiple patient subsets are required.

The limitations of this study include a relatively small sample size which consists of ICI monotherapy and ICIs plus chemotherapy. When we only analyzed ICIs plus chemotherapy cohort, we also found association between early changes in ctDNA levels with the clinical response (*p* = 0.024, Figure S5A). Although there was no significant survival benefit in patients with over a 50% reduction (PFS, *p* = 0.061; OS, *p* = 0.054), the trend was obvious (Figure S5B). Thus, we will enroll a greater number of patients for further validation in future study.

In conclusion, we performed tumor‐informed assay for the evaluation of ctDNA to predict the response to first‐line immunotherapy in patients with locally advanced or metastatic NSCLC. Our findings suggest that tumor‐informed assay is a better option for ctDNA detection, and early dynamic changes in plasma ctDNA may represent a valuable biomarker for monitoring the efficacy and predicting the outcome in advanced NSCLC patients treated with first‐line ICIs ± chemotherapy.

## AUTHOR CONTRIBUTIONS

**Lei Cheng:** Conceptualization (equal); data curation (equal); formal analysis (equal); writing – original draft (equal); writing – review and editing (equal). **Guanghui Gao:** Conceptualization (equal); data curation (equal); writing – review and editing (equal). **Chao Zhao:** Conceptualization (equal); data curation (equal); writing – review and editing (equal). **Haowei Wang:** Data curation (equal). **Chao Yao:** Methodology (equal); visualization (equal). **Hanchuanzhi Yu:** Methodology (equal); visualization (equal). **Jicheng Yao:** Methodology (equal); visualization (equal). **Feng Li:** Data curation (equal); methodology (equal). **Lijie Guo:** Visualization (equal). **Qijie Jian:** Visualization (equal). **Xiaoxia Chen:** Conceptualization (equal); writing – review and editing (equal). **Xuefei Li:** Conceptualization (equal); funding acquisition (equal); project administration (equal); writing – review and editing (equal). **Caicun Zhou:** Conceptualization (equal); funding acquisition (equal); project administration (equal).

## FUNDING INFORMATION

This study was partly supported by grants from the National Natural Science Foundation of China (No. 81972169), Shanghai Municipal Health Commission Project (Shanghai Key Clinical Specialty Construction Project ‐ Respiratory Medicine), Shanghai Municipal Health Commission Project (No. 2020CXJQ02), and Shanghai Hospital Development Center (No. SHDC2020CR1036B).

## CONFLICT OF INTEREST STATEMENT

No potential conflicts of interest are disclosed.

## Supporting information


Figure S1.

Figure S2.

Figure S3.

Figure S4.

Figure S5.

Table S1.

Table S2.

## Data Availability

The datasets generated in the current study are available from the corresponding author on reasonable request.
