# Supplementary figures and images for: Personalized circulating tumor DNA detection to monitor immunotherapy efficacy and predict outcome in locally advanced or metastatic non‐small cell lung cancer
Source: Cancer Med. 2023 May 15;12(13):14317–26. doi: 10.1002/cam4.6108 (PMC10358227; doi:10.1002/cam4.6108)

**A**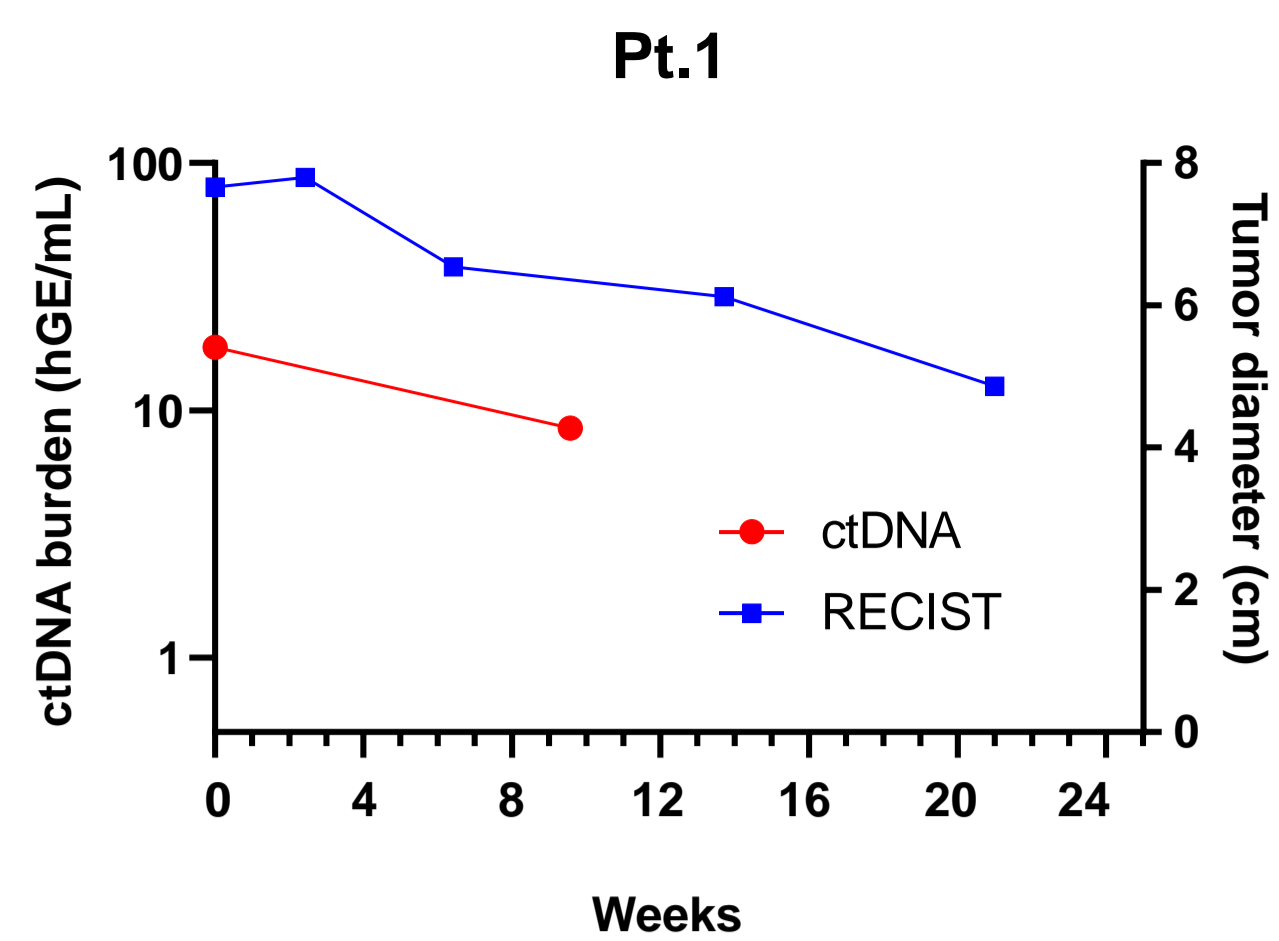**B**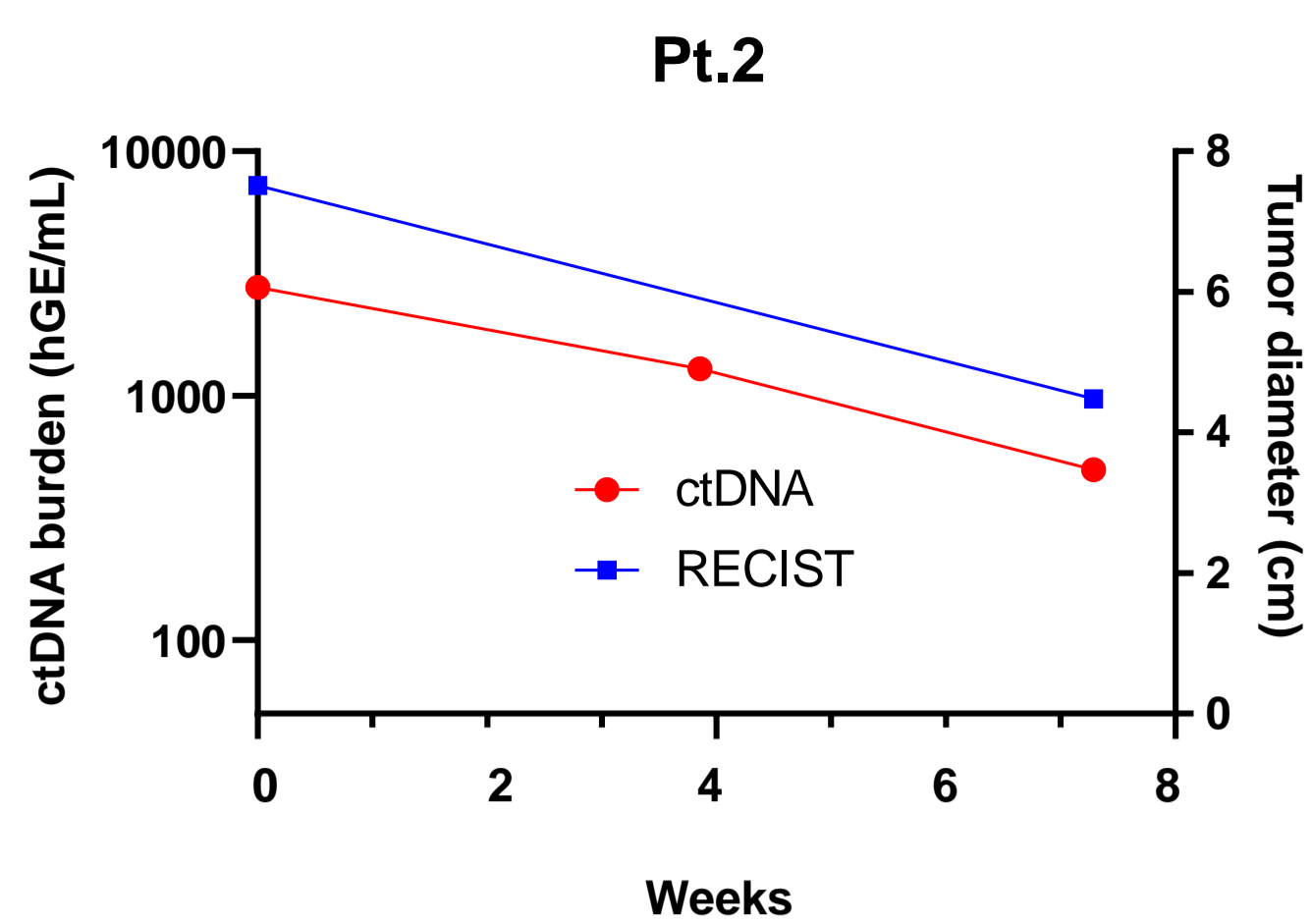**C**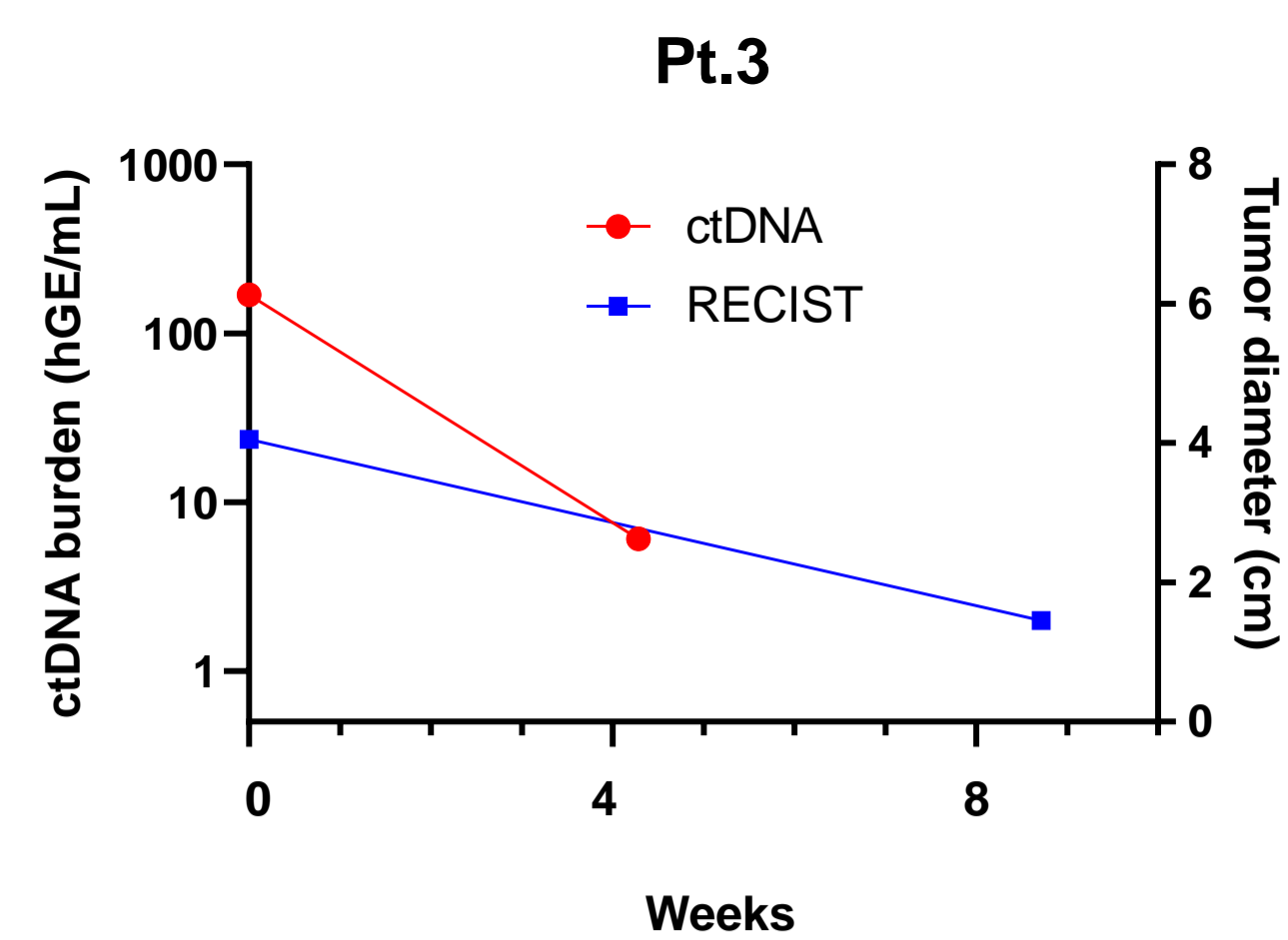**D**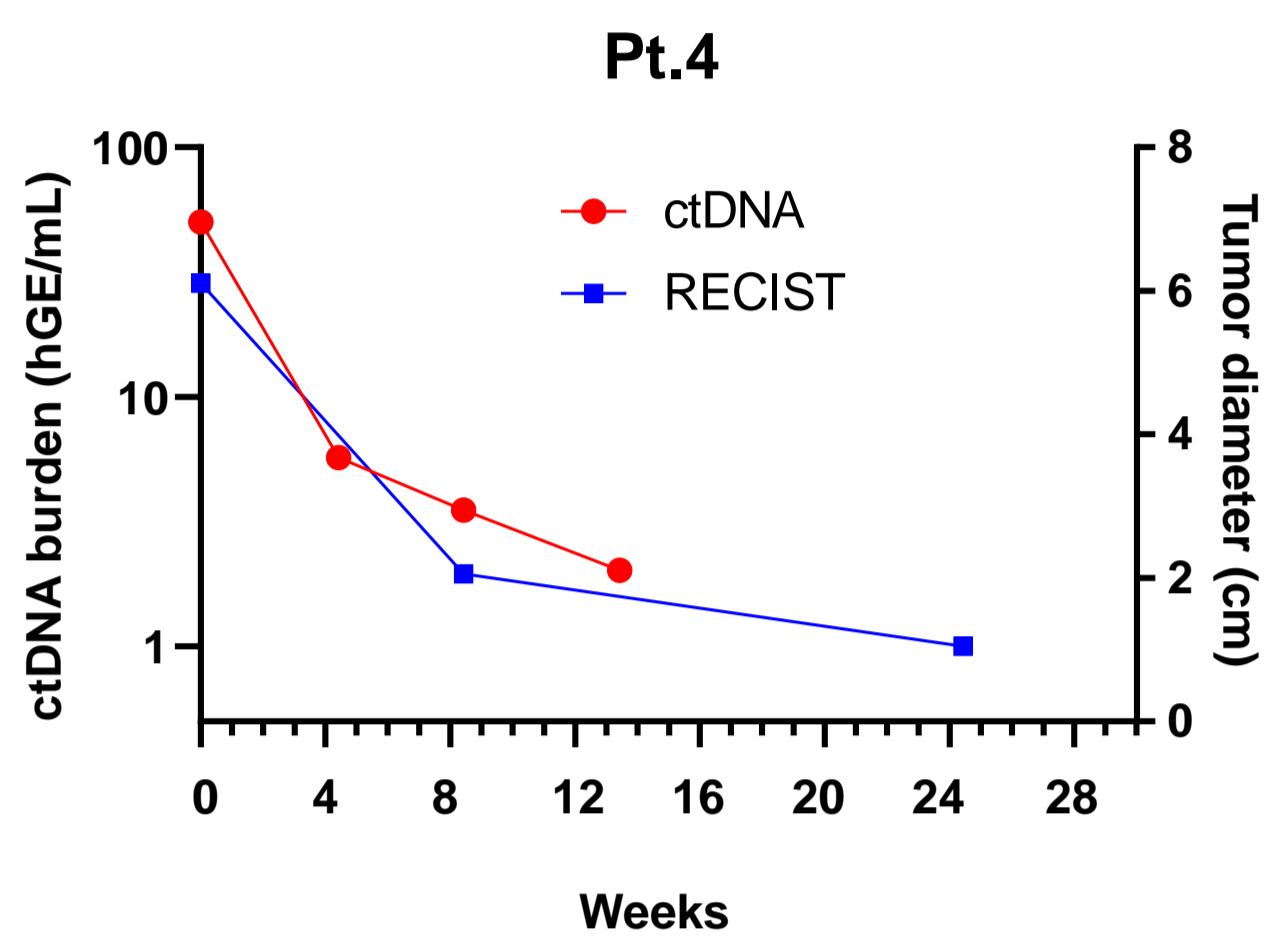**E**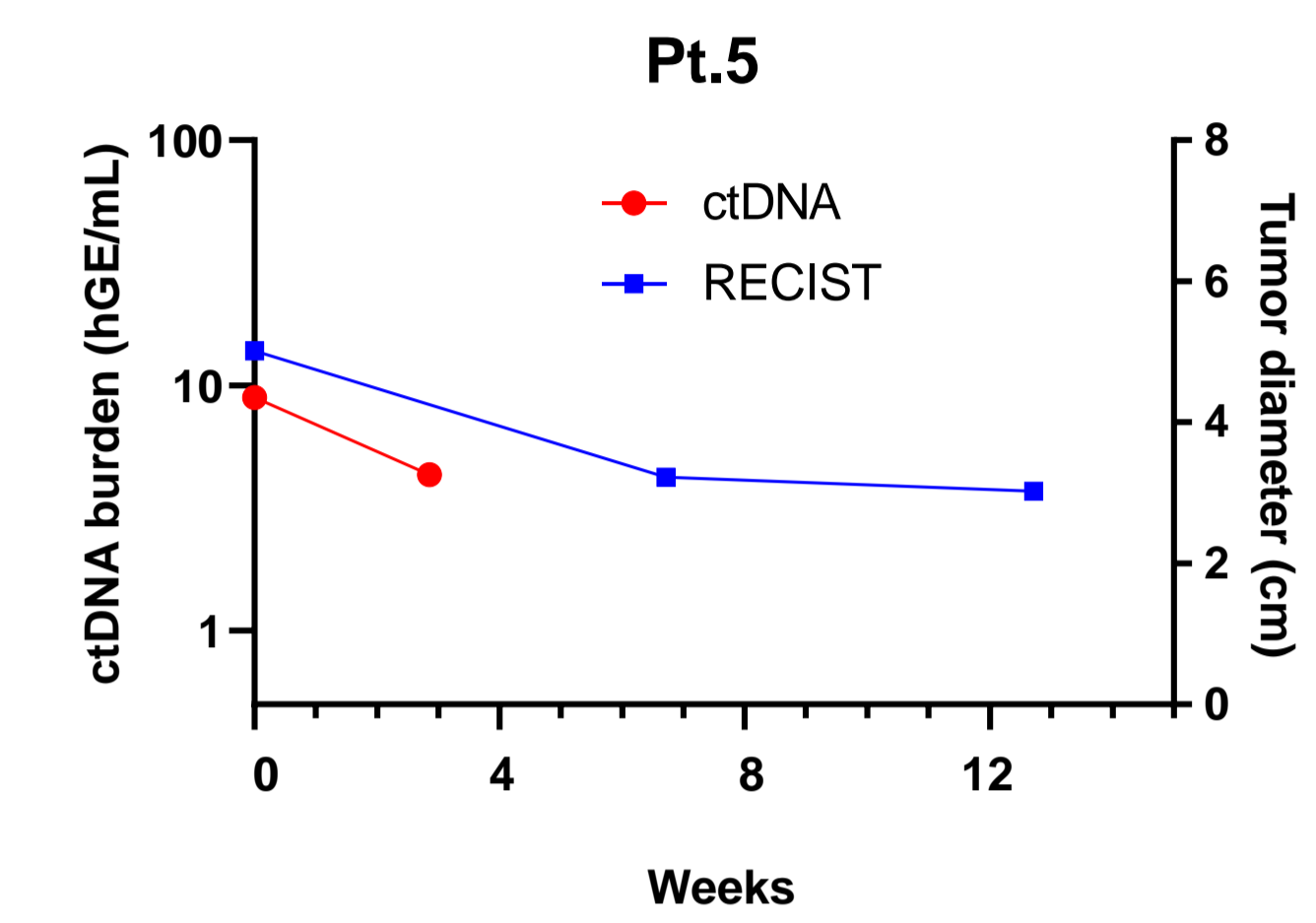**F**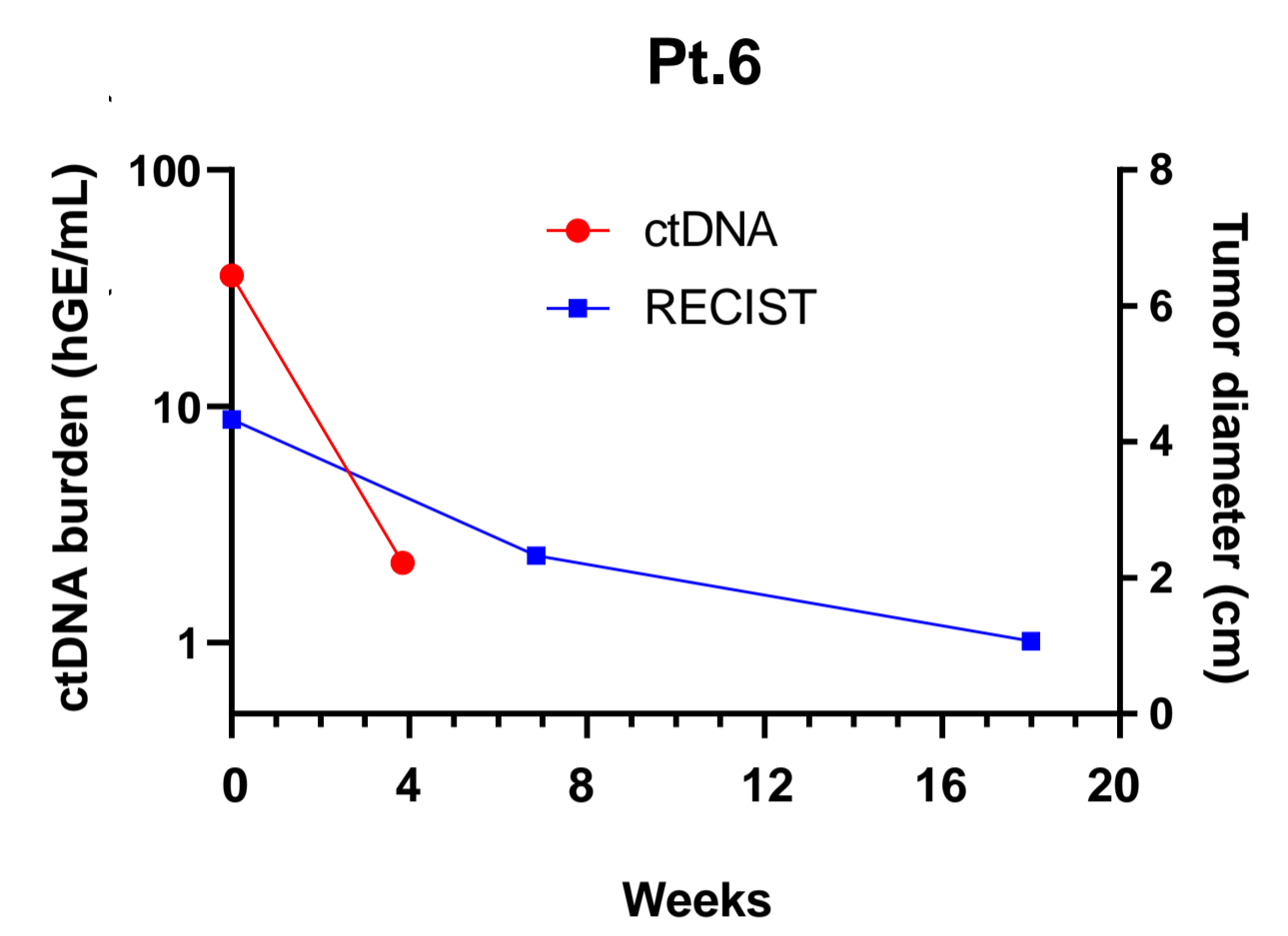**G**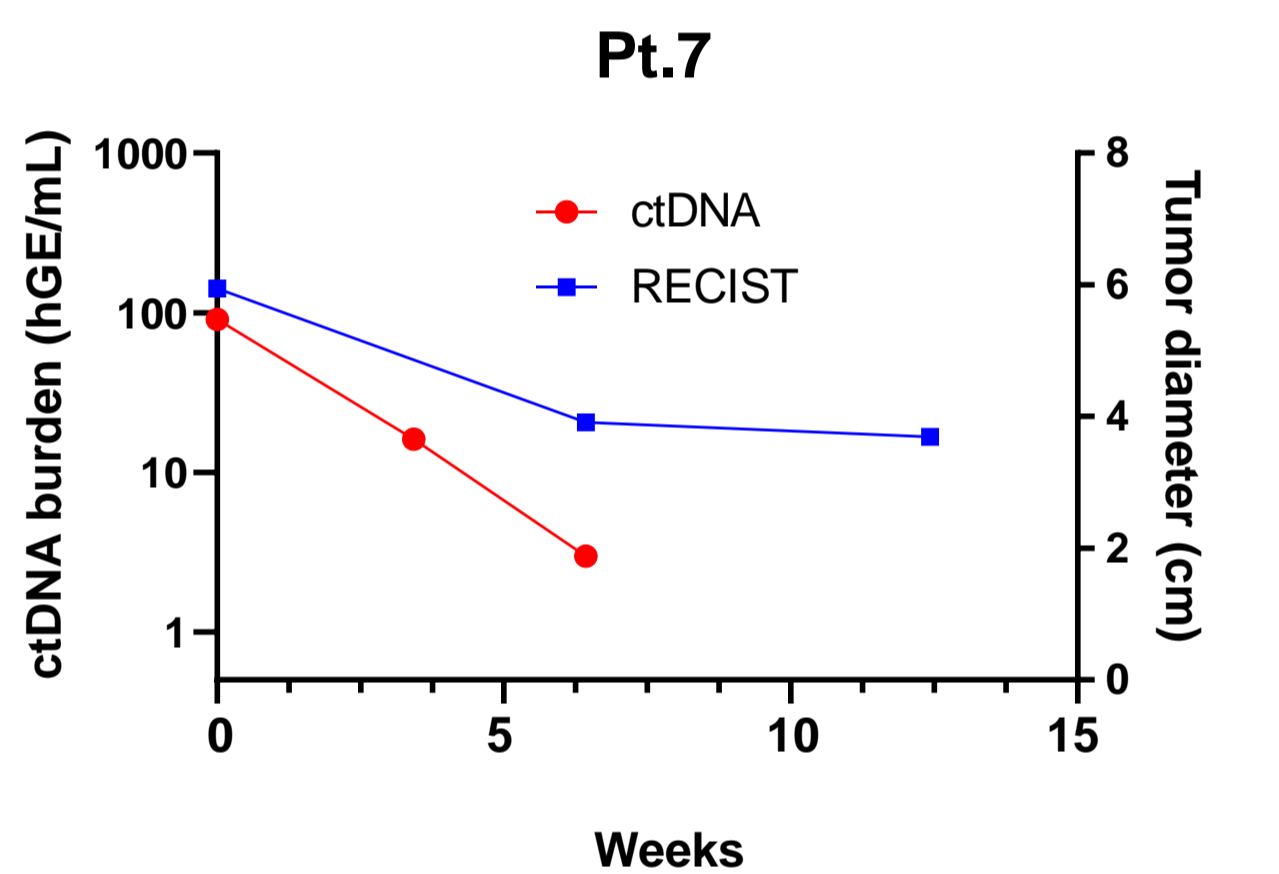**H**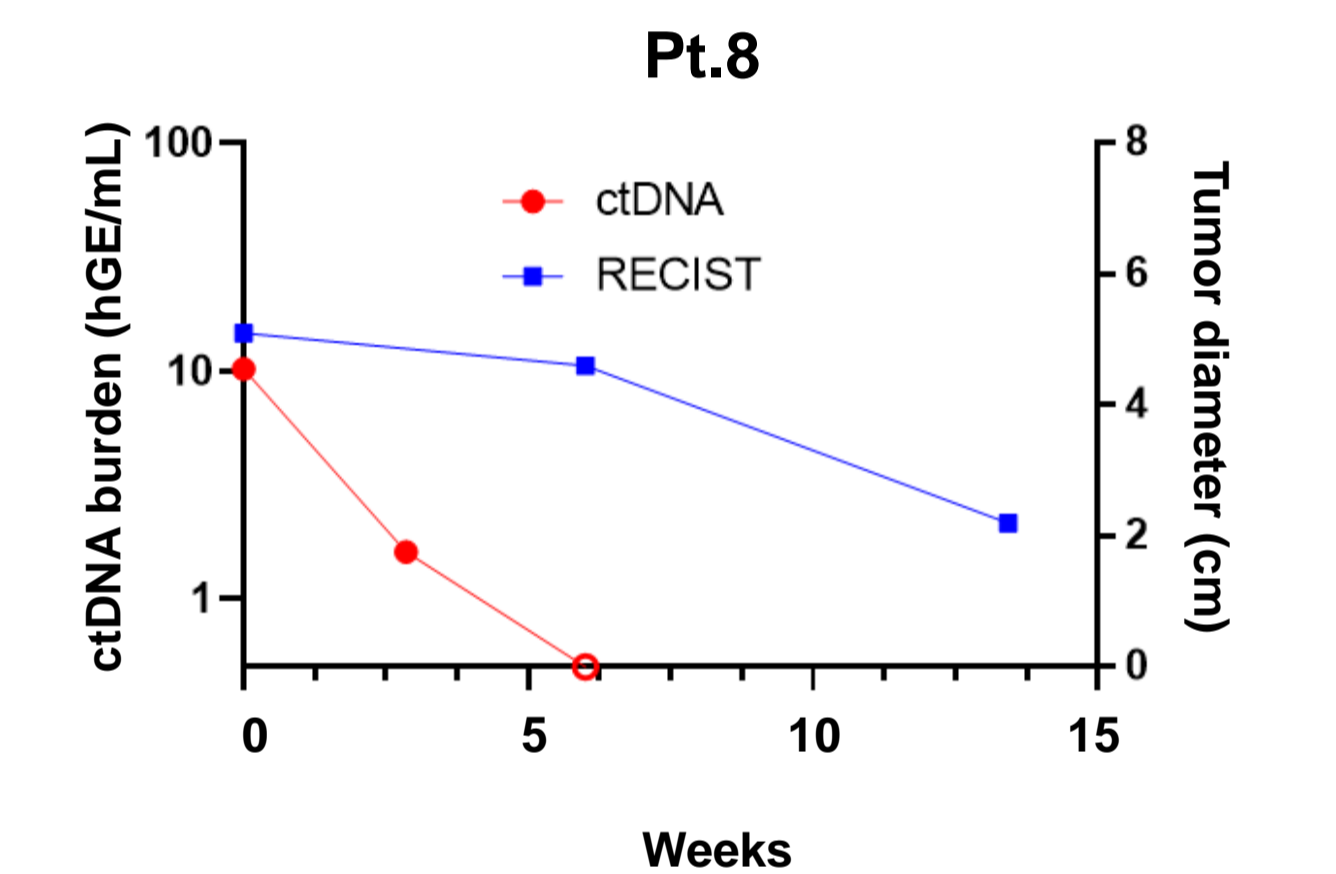**I**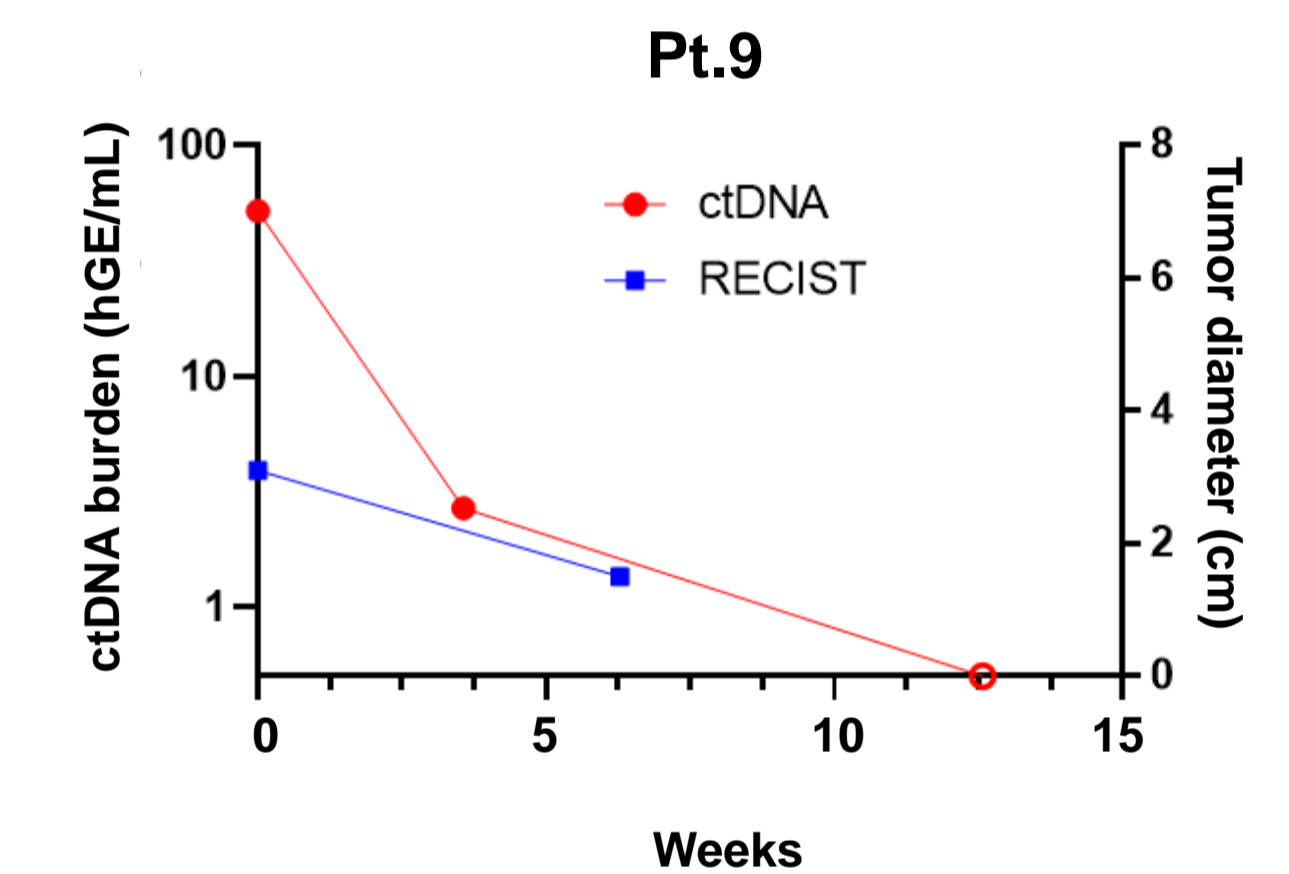**J**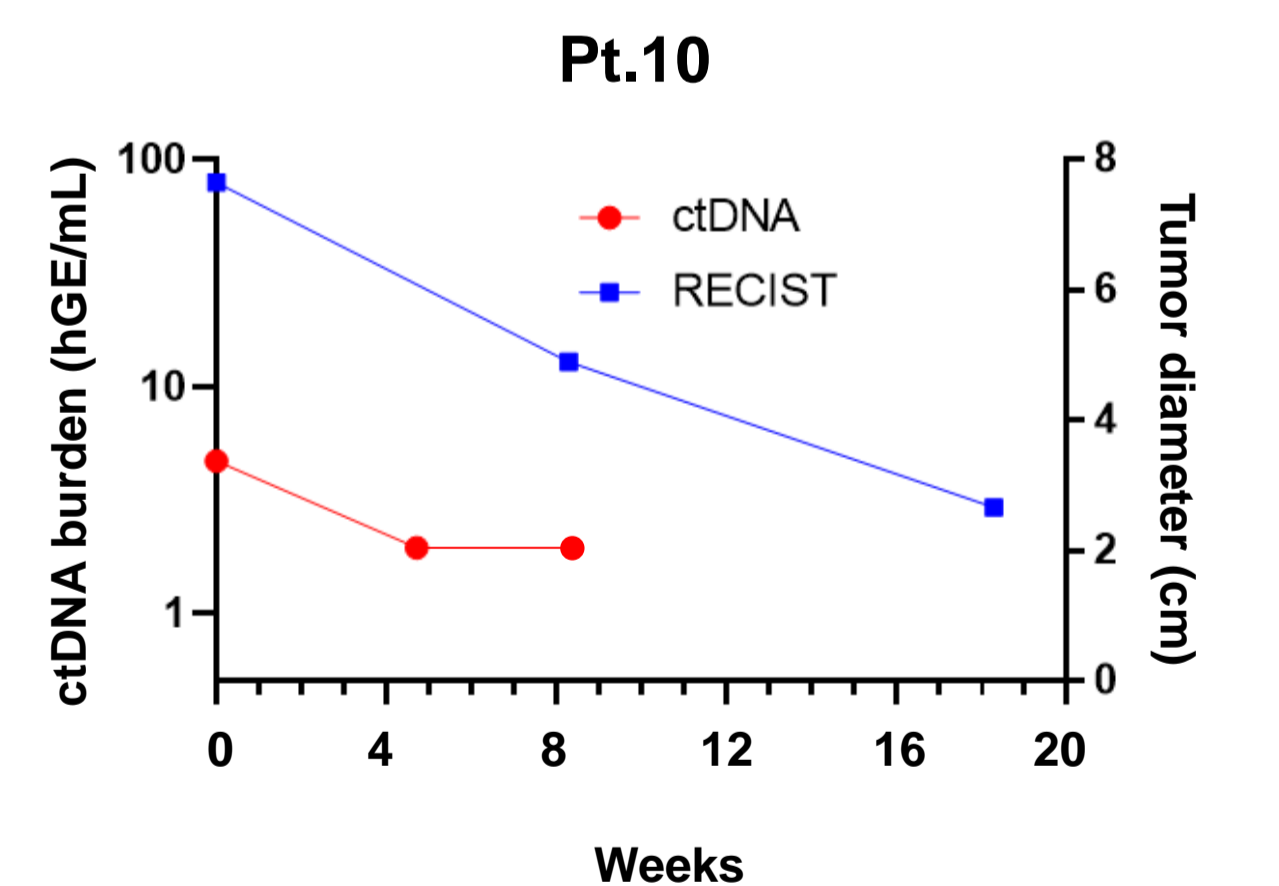**K**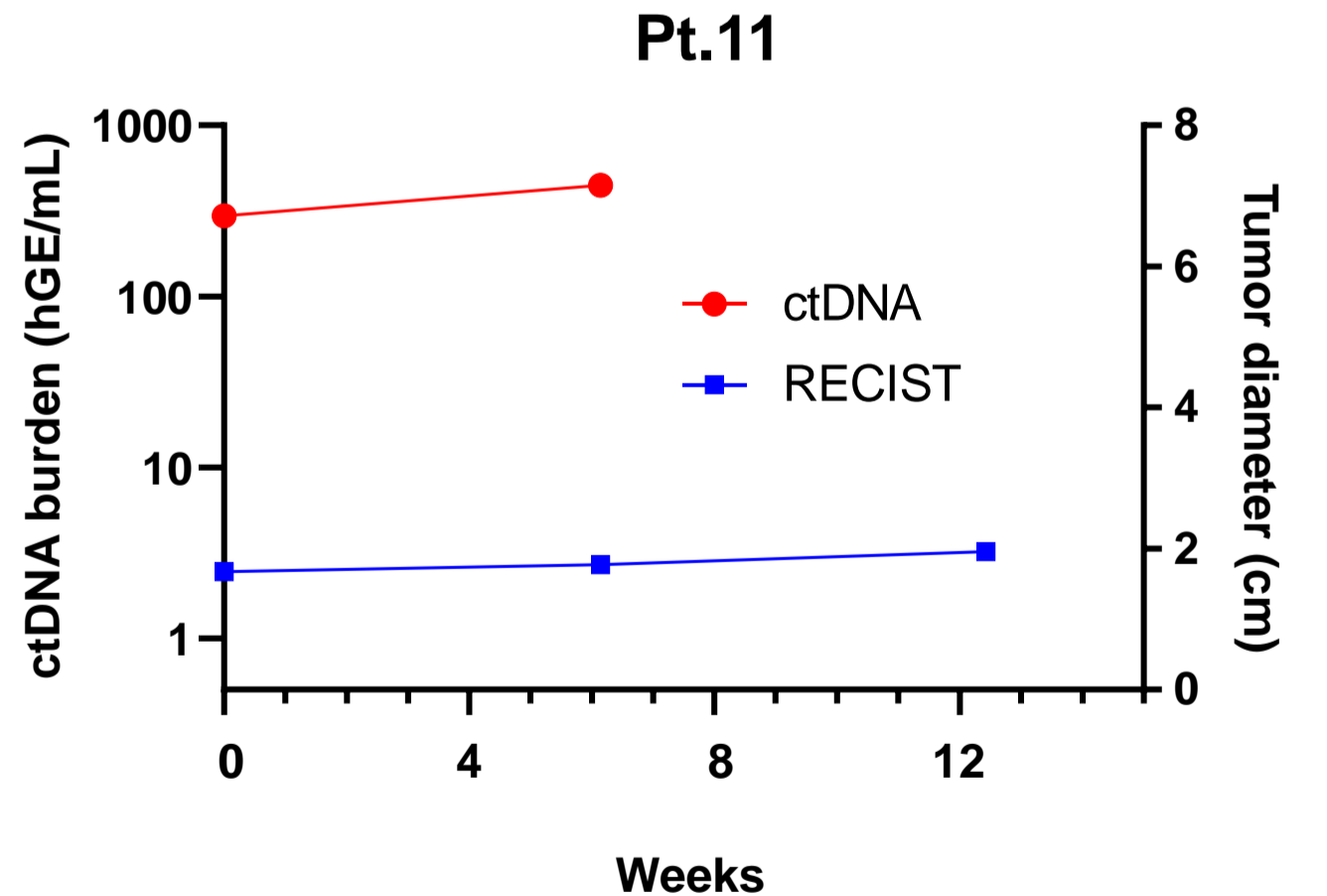**L**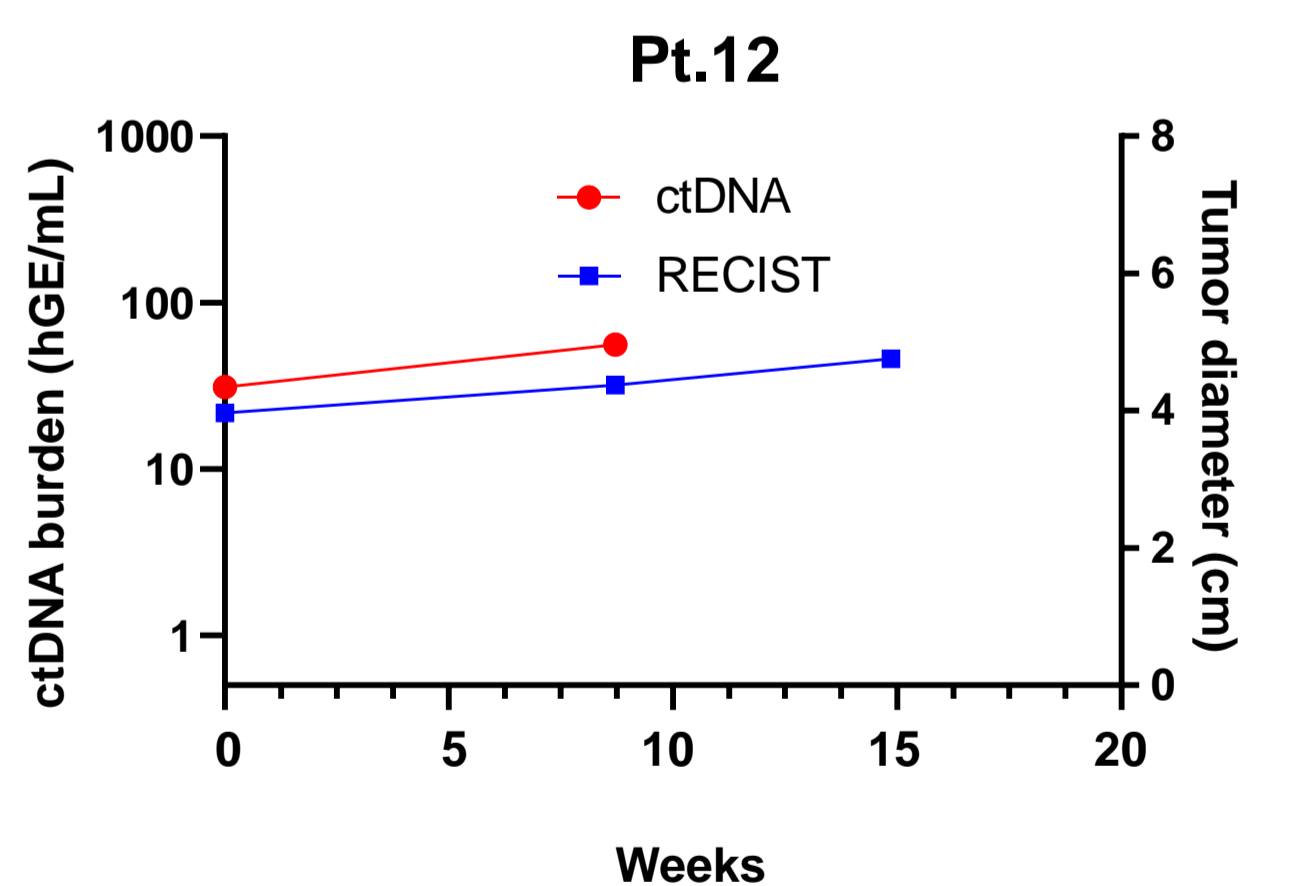**M**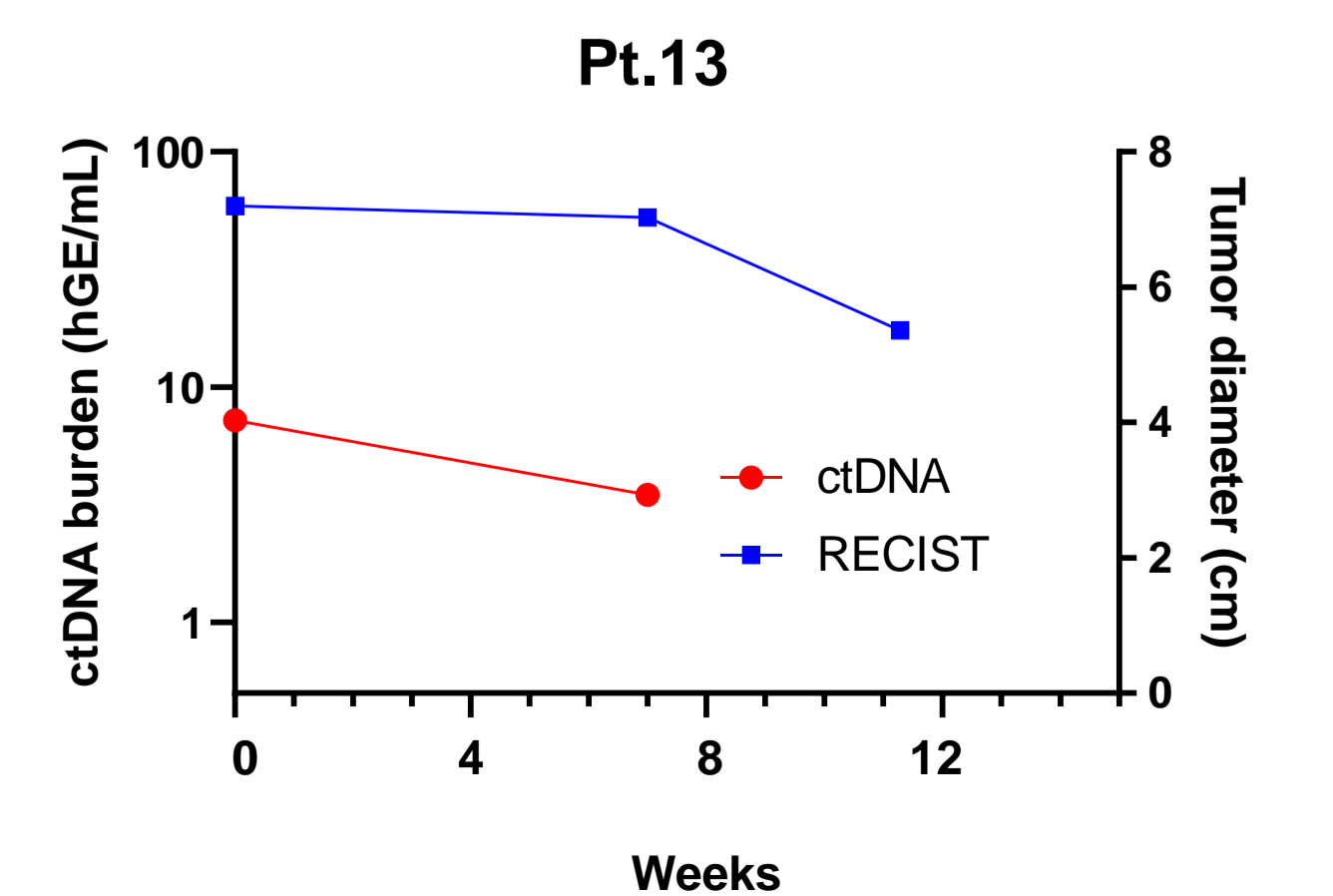

Supplement: Supplementary file 1 — Figure S1. [file CAM4-12-14317-s002.pdf]

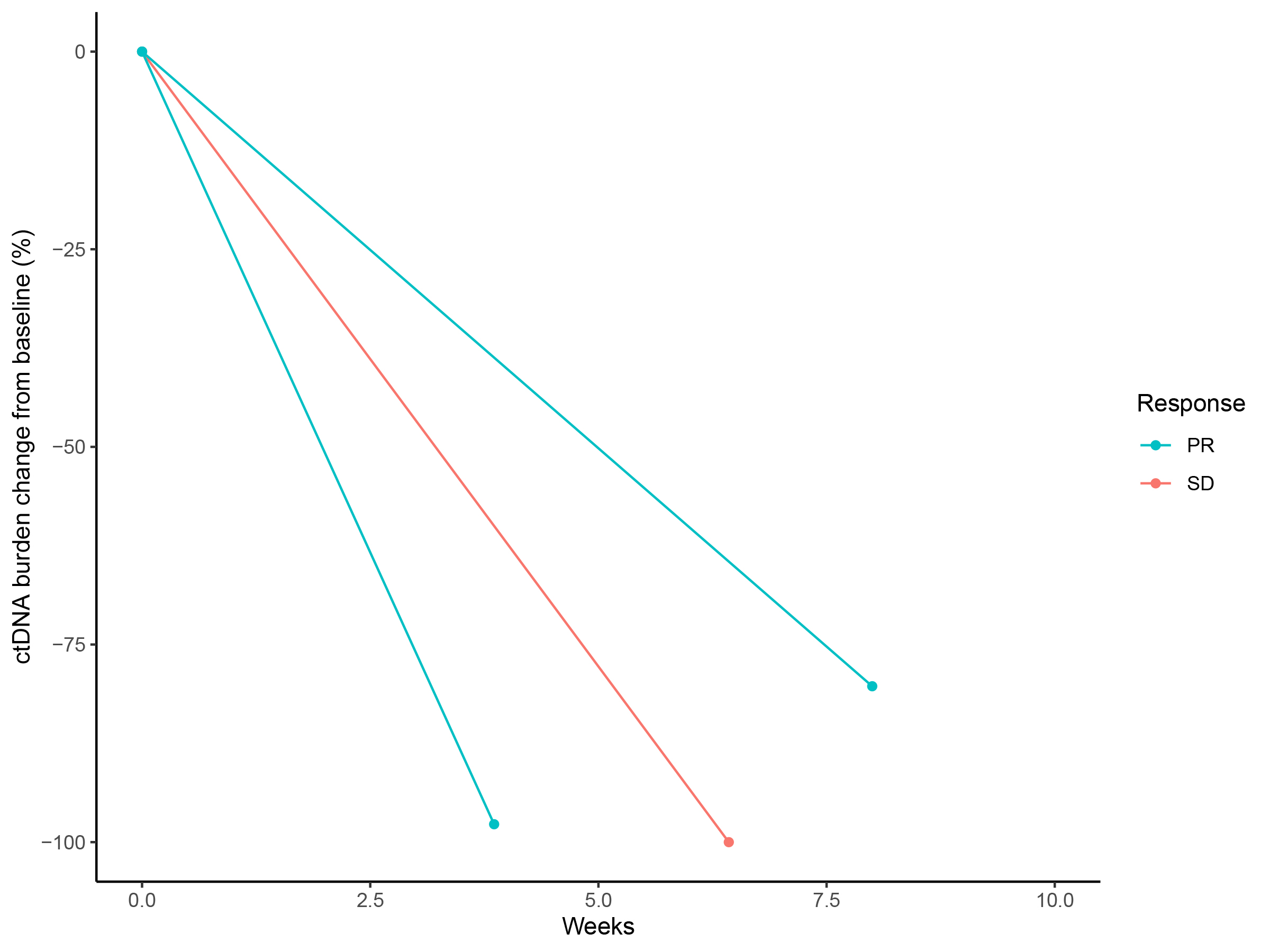

Supplement: Supplementary file 2 — Figure S2. [file CAM4-12-14317-s001.jpg]

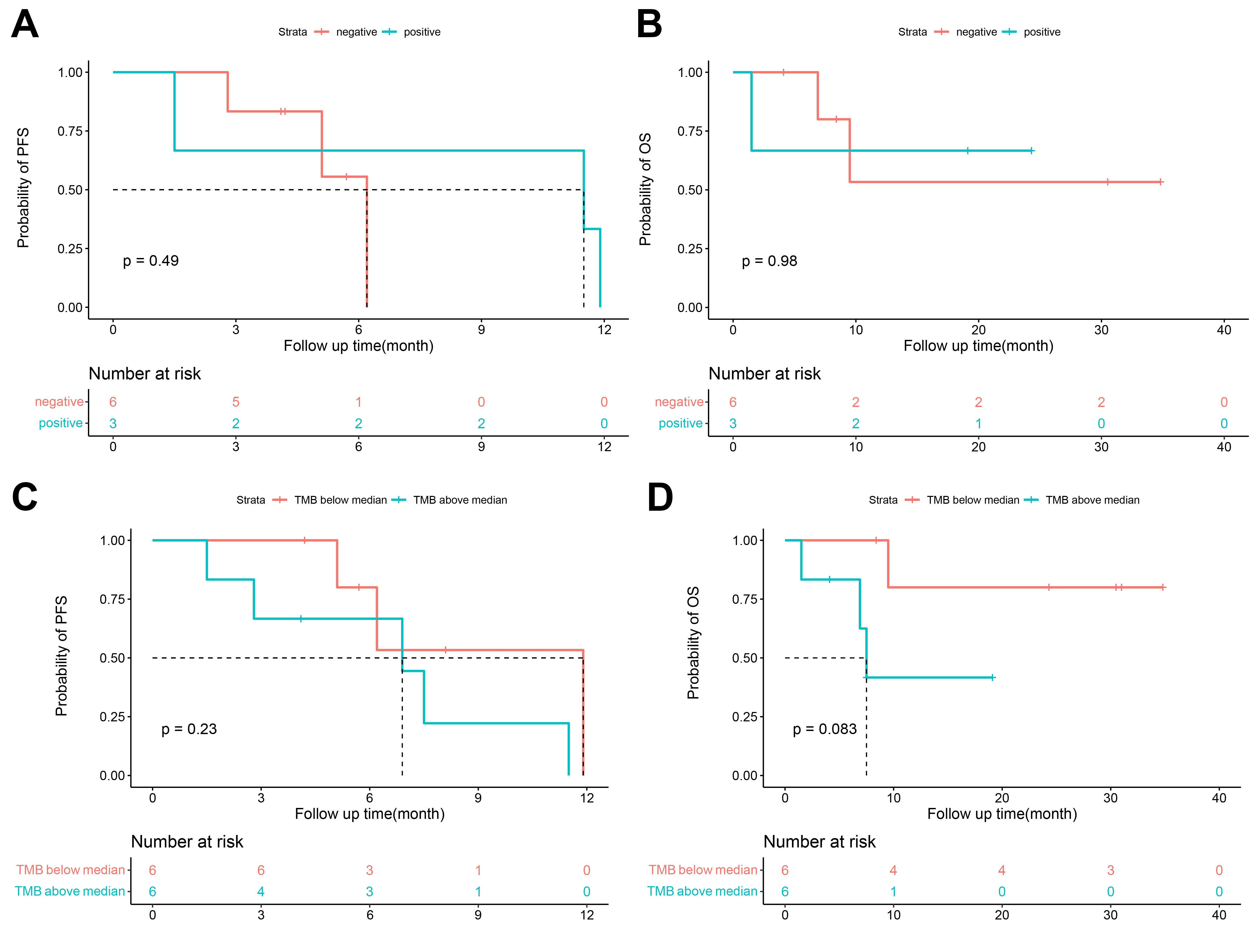

Supplement: Supplementary file 3 — Figure S3. [file CAM4-12-14317-s005.jpg]

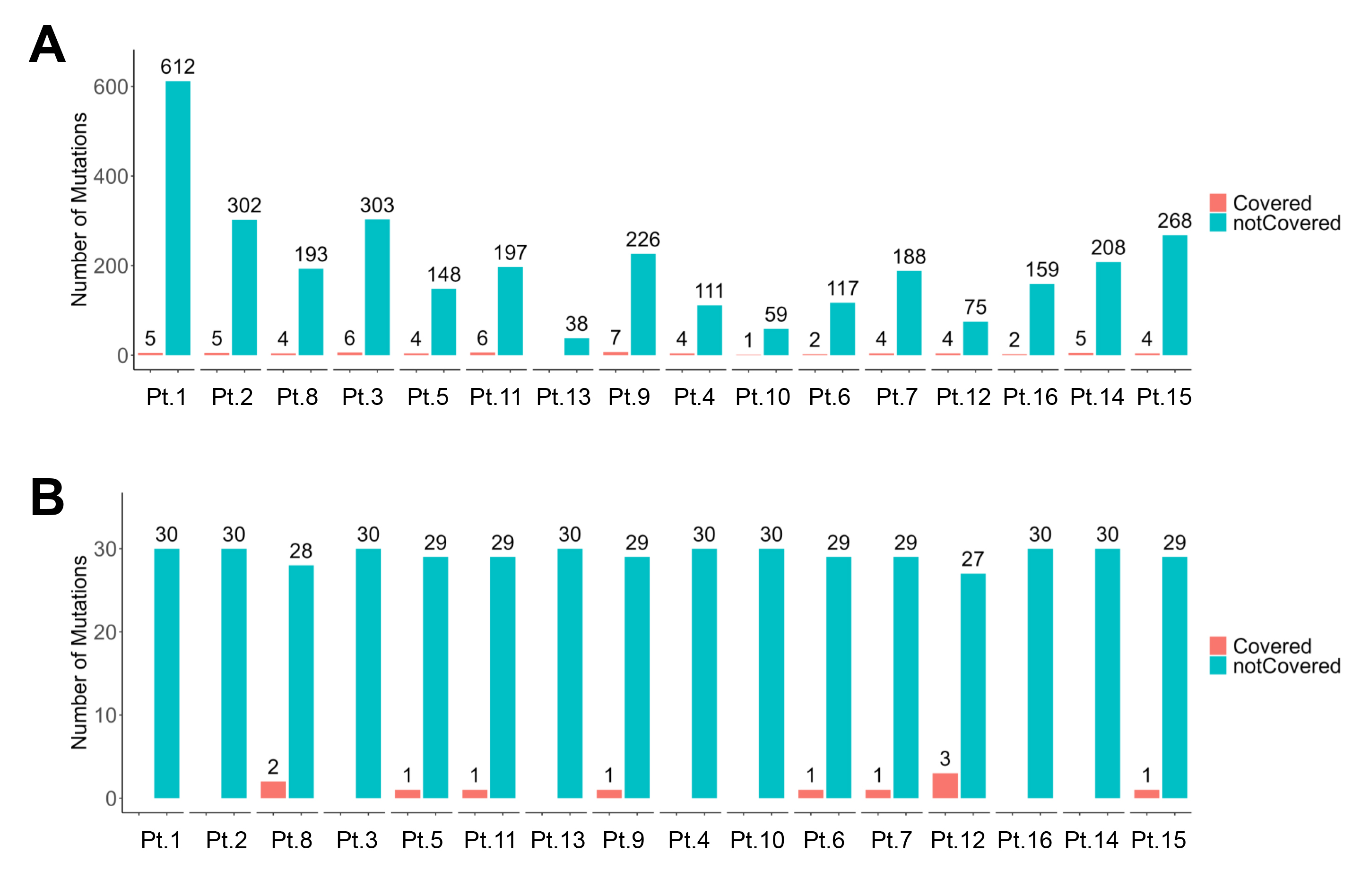

Supplement: Supplementary file 4 — Figure S4. [file CAM4-12-14317-s003.jpg]

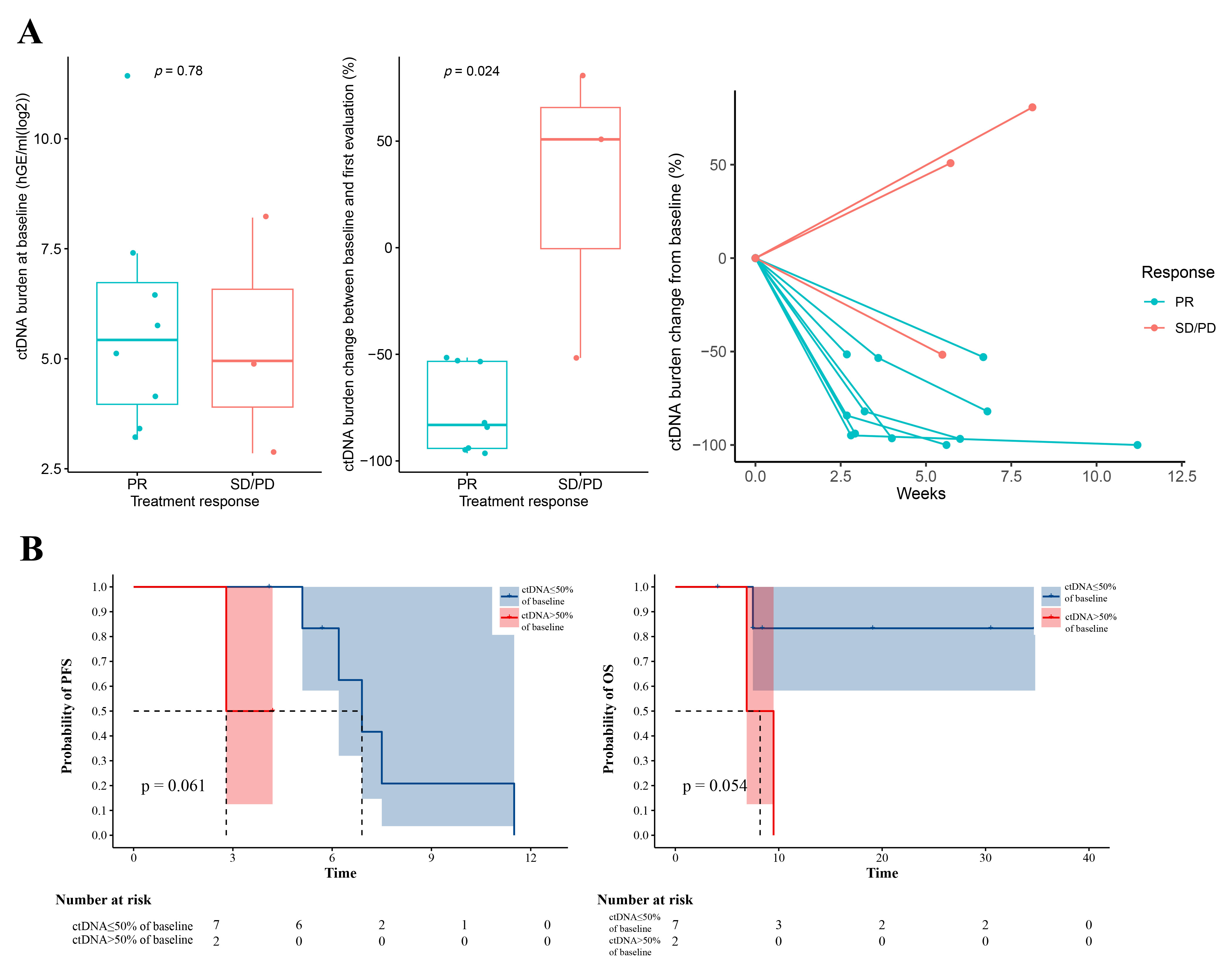

Supplement: Supplementary file 5 — Figure S5. [file CAM4-12-14317-s007.tif]
