# Supplementary material for: Personalized circulating tumor DNA detection to monitor immunotherapy efficacy and predict outcome in locally advanced or metastatic non‐small cell lung cancer
Source: Cancer Med. 2023 May 15;12(13):14317–26. doi: 10.1002/cam4.6108 (PMC10358227; doi:10.1002/cam4.6108)
Supplement: Supplementary file 7 — Table S2. [file CAM4-12-14317-s006.docx]

Supplementary Table 2 The ctDNA detection analysis using a patient-specific panel designed based on cfDNA WES.

| **Patients** | **SNVs detected by cfDNA WES** | | **SNVs** **designed** | **SNVs detected by blood-informed assay** | |
| --- | --- | --- | --- | --- | --- |
|  | No. | VAF | No. | No. | VAF |
| Pt.17 | 106 | 1.79% | 27 | 26 | 1.30% |
| Pt.18 | 19 | 1.64% | 14 | 14 | 1.86% |
| Pt.19 | 49 | 5.57% | 37 | 37 | 6.39% |

ctDNA, circulating tumor DNA. cfDNA, cell-free DNA. WES, whole-exome sequencing. SNV, single nucleotide variant. VAF, variant allele fraction.
